# A new method and insights for estimating phenological events from herbarium specimens

**DOI:** 10.1002/aps3.1224

**Published:** 2019-03-07

**Authors:** Katelin D. Pearson

**Affiliations:** ^1^ Department of Biological Science Florida State University 319 Stadium Drive Tallahassee Florida 32306 USA

**Keywords:** climate change, digitization, herbarium specimens, phenology

## Abstract

**Premise of the Study:**

A novel method of estimating phenology of herbarium specimens was developed to facilitate more precise determination of plant phenological responses to explanatory variables (e.g., climate).

**Methods and Results:**

Simulated specimen data sets were used to compare the precision of phenological models using the new method and two common, alternative methods (flower presence/absence and ≥50% flowers present). The new “estimated phenophase” method was more precise and extracted a greater number of significant species‐level relationships; however, this method only slightly outperformed the simple “binary” (e.g., flowers present/absent) method.

**Conclusions:**

The new method enables estimation of phenological trends with greater precision. However, when time and resources are limited, a presence/absence method may offer comparable results at lower cost. Using a more restrictive approach, such as only including specimens in a certain phenophase, is not advised given the detrimental effect of decreased sample size on resulting models.

Plant phenology—the timing of life history events such as flowering or fruiting—is a key aspect of plant fitness (Inouye, 2008) and the study of climate change (Menzel, 2002). Pressed, preserved plant specimens (“herbarium specimens”) collected over centuries and around the globe can serve as snapshots of plant phenology and have contributed to a wealth of research relating the timing of phenological events to, e.g., climate change (Willis et al., 2017a; Jones and Daehler, 2018), disturbance (Gómez‐Garcia et al., 2009), plant traits (Bolmgren and Lönnberg, 2005), and climate seasonality (Sahagun‐Godinez, 1996). Although most herbarium specimens were not collected with the purpose of estimating phenological events, phenological data from specimens have proven reliable and irreplaceable for understanding plant phenology (Davis et al., 2015).

In most studies of phenology using herbarium specimens, the timing of a phenological event is determined from the collection date and some assessment of the “phenophase” (i.e., phase within a longer phenological event, such as peak flowering) of one or many specimens. For example, the majority (64%) of flowering studies reviewed in Willis et al. (2017a) examined phenological trends using only specimens that had open flowers. Other authors have simply assumed that the collection date of the specimen corresponds to flowering date (Gaira et al., 2014; Pei et al., 2015; Munson and Long, 2017), because specimens are most often collected when reproductive. Several authors (14% of flowering studies reviewed in Willis et al., 2017a) have taken a more selective approach by including only specimens upon which 50% or even 75% (Davis et al., 2015) of reproductive structures are open (sensu Primack et al., 2004). In each of these methods, the collection dates of the selected specimens served as the response variable, while some other variable (e.g., climate or year) acted as the explanatory variable, producing a phenological relationship between these two variables in the form of a slope (e.g., days earlier or later flowering/°C).

These heuristic methods enable fairly rapid assessment of specimen phenophases and have proven sufficient to elucidate general phenological trends across great spatiotemporal scales; however, these approaches have limitations. The “binary method” (i.e., including all specimens with a certain reproductive structure present) may produce imprecise estimates of phenology because phenological events may span weeks or months (Panchen and Gorelick, 2017). When these estimates are used to relate phenology and an explanatory variable (e.g., temperature), the resulting relationship may be inaccurate due to the high variance (Fig. 1A) or imprecise, reducing confidence in its estimated value. The “≥50% method” (including only specimens with 50% or more of reproductive structures in a certain state, such as open flowers) may improve the estimation of relationships between phenology and explanatory variables—albeit moderately—by allowing comparison of specimens in more similar phenophases (Fig. 1B). Nevertheless, this method may limit sample size because a significant proportion of specimens may be reproductive yet fail to meet the inclusion criterion. Larger sample sizes generally increase confidence in modeled relationships, which may be particularly important for the often “noisy” data produced by specimen records. Phenological data are subject not only to the vagaries of specimen collection but also to the subjectivity of human phenophase determinations, adding additional error to estimates of phenological events and highlighting the need for a more precise approach (e.g., Willis et al., 2017b).

**Figure 1:**
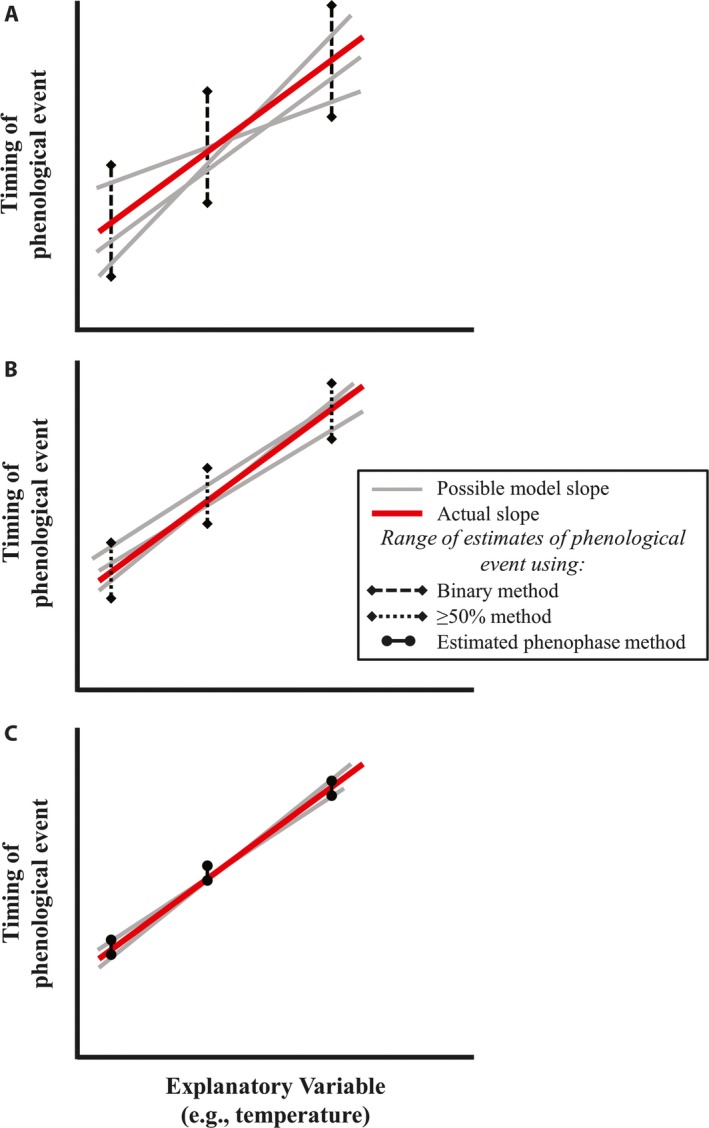
Graphical representation of how the binary (A), ≥50% (B), and estimated phenophase (C) methods estimate the relationship between the timing of a phenological event and an explanatory variable. The binary method (A) uses the collection date of all specimens in any phase within the longer phenological event (e.g., at least one flower present). Thus, the span of time within which the collection date can occur is potentially as long as the flowering season. When compounded, these imprecise phenological approximations can lead to erroneous estimates of the relationship between phenology and the explanatory variable or even preclude discovery of the relationship. Similarly, the range of possible dates for the ≥50% method (B) may be wide if the duration of peak flowering is long, and estimates of phenological relationships may be accordingly imprecise. In contrast, the estimated phenophase method (C) can theoretically provide a more precise estimate of the phenological relationship by enabling comparison of a specific point within the phenological event among values of the explanatory variable.

This paper describes a novel “estimated phenophase” method that aims to overcome these limitations. In this method, the relative phenophase of each specimen within the duration of the phenological event is considered, and a more precise, comparable metric is estimated using the collection date and in situ, observational phenophase duration data. The phenological event discussed in this paper is flowering, and the estimated metric is peak flowering date; however, the general premise of the study—estimating a specific phase from within a phenological event—could be applied to fruiting, leaf‐out, or other phenological events.

Coupling specimen‐based and observational data produces a metric that potentially allows for a more precise relation of phenological events to explanatory variables (Fig. 1C) and maximizes the number of specimens that can be included in the model data set. Here I use simulated specimen data sets to compare the performance of phenological models built using the binary, ≥50%, and estimated phenophase specimen inclusion methods.

## METHODS AND RESULTS

### Estimated phenophase method

The estimated phenophase method was originally developed for a phenological study of 81 species in 11 asteraceous genera in the southeastern United States (Pearson, 2019). It would be impossible to evaluate the accuracy and precision of actual data without knowing the “true” values for phenological events. Therefore, the data evaluated here are solely from simulations, and only the general methodology for collecting specimen‐based and observational data is described here.

In the study of southeastern U.S. Asteraceae, flowering phenology was assessed from herbarium specimen images downloaded via the iDigBio portal (https://www.idigbio.org/portal/search); however, the method can be employed more generally to score physical specimens or specimen images. Each specimen or specimen image was assigned a numerical phenophase from 1–9 based on the percentage of buds, flowers, and fruits present on the specimen using the nearest quartile values (0%, 25%, 50%, 75%, or 100%) such that the total of all reproductive structures on a specimen equaled 100%. For example, specimens on which 100% of reproductive structures were buds were assigned phenophase 1, specimens with 75% buds and 25% flowers were assigned phenophase 2, and so on (see Appendix 1 for detailed schema). Examples of specimens with their assigned phenophases are also shown in Appendix 1.

The peak flowering date of each specimen was estimated by adding or subtracting days from the collection day of year (DOY; 1–365), determined from observational flowering duration data as follows. Wild populations of one species from each focal plant genus were identified in Leon County, Florida, USA, prior to or near the beginning of their flowering periods, and several (11–25) individuals of each species were marked. In this study, sample size was limited by plant availability and resources, and larger sample sizes are recommended for future applications of this method. The quartile percentages of buds, flowers, and fruits on each plant were recorded every 3–4 days until the end of the flowering period (i.e., 100% fruits), and these percentages were converted into phenophases following the same schema applied to herbarium specimens (see Appendix 1). For each species, a linear mixed effects model (*lmer* function of *lme4* package in R; R Core Team, 2016) was used to determine the number of days elapsed (continuous fixed effect) per phenophase, taking into account different starting dates of individuals (random effect). The slope of this model (days per phenophase) was used to adjust the day of flowering for each specimen record to reflect estimated date of peak flowering (phenophase 5). For example, the estimated duration of each phenophase in the genus *Coreopsis* L. was 1.7 days; thus the date of peak flowering for a *Coreopsis* specimen in phenophase 8 would be estimated by subtracting 5.1 days (1.7 days/phenophase × [8 − 5] phenophases) from the collection DOY.

This method operates under the following assumptions: (1) the relationship between time and phenophase is reasonably linear, (2) flowering duration does not significantly co‐vary with the explanatory variable or other potentially confounding variables (e.g., latitude), and (3) flowering duration is similar among species within a genus. These assumptions were considered reasonable in the study of 11 asteraceous genera in the southeastern United States (Pearson, 2019), but this may not be the case for other regions or taxa. Although there is substantial evidence that flowering time (e.g., date of first flowering) varies with latitude (e.g., Weber and Schmid, 1998) and may be phylogenetically conserved in some taxa (e.g., Davies et al., 2013), there are few data on the effect of these factors on flowering duration (but see Wagner and Simons, 2009, which supports assumptions 1 and 2). Therefore, the assumptions listed above must be checked when using the estimated phenophase method. Still, if one or more assumptions prove invalid, the method could be easily modified. For example, if the relationship between time and phenophase is non‐linear (i.e., assumption 1 is invalid), any number of alternative models (e.g., curvilinear) could be used to calculate the number of days to add or subtract from the collection date to estimate peak flowering date. If flowering duration is suspected to differ between species within a genus (i.e., assumption 3 is invalid), the phenology of multiple species can be monitored in the field, provided the species and resources are available.

### Specimen data set simulations

**Figure 2:**
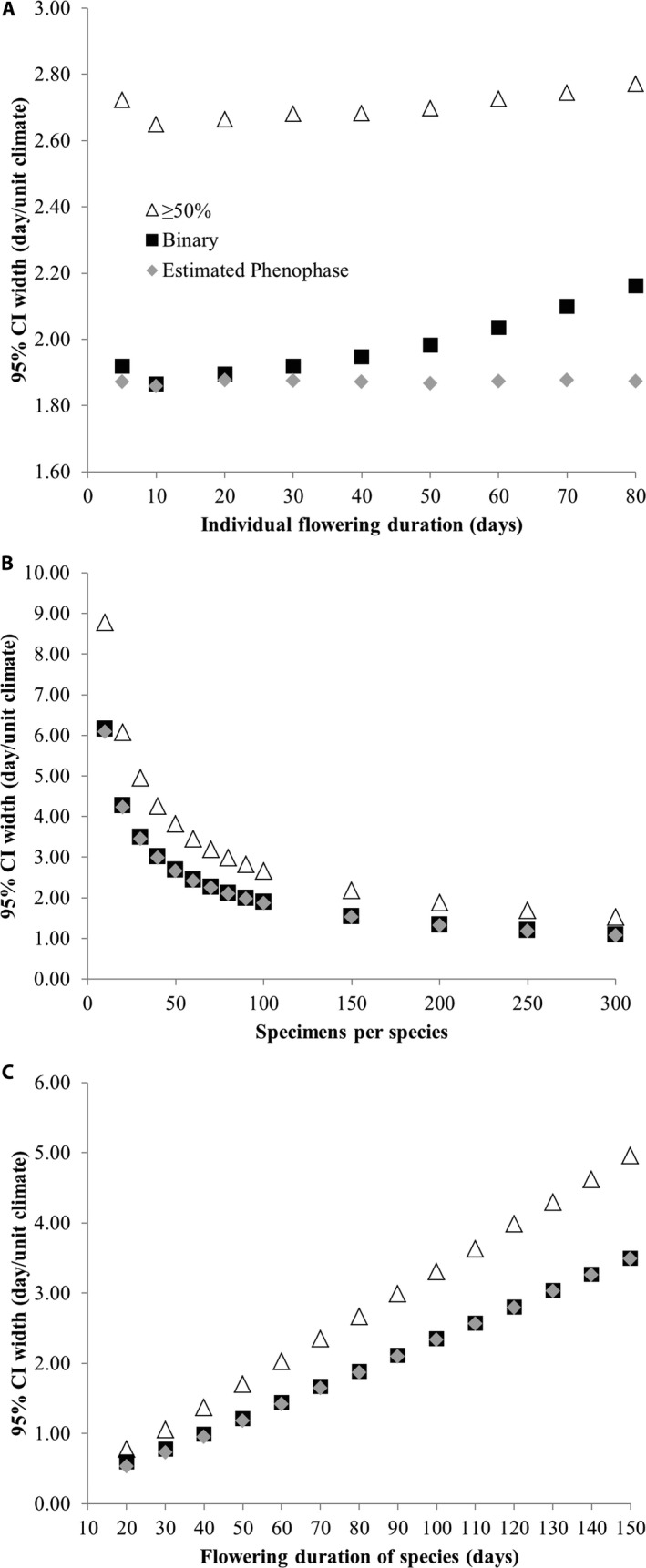
Width of 95% confidence intervals (CIs) of slope (days/unit climate) estimated using the ≥50% (white triangles), binary (black squares), and estimated phenophase (gray circles) methods of specimen inclusion with changes in key simulation parameters. For simplicity, results from linear mixed effects (LME) models in which only species intercepts were allowed to vary are shown; variable intercept + slope model results are provided in Appendix S3. Each point represents the mean value of 100 iterations of the simulation. Standard errors of the mean (listed in Appendix S2) were very small and are not included in this figure for clarity. (A) The length of the individual flowering duration was changed between simulations. (B) The number of specimens per species was varied while keeping all other variables, including number of species, constant. (C) 95% CI widths are shown with increasing flowering season durations of species. Unless otherwise specified, default simulation parameters were as described in Table 1.

Simulated specimen data sets were created using custom R code (Appendix S1) to evaluate how well the estimated phenophase method resolved relationships between phenology and a theoretical “climate” variable relative to the binary and ≥50% flowering methods. For each iteration, the simulation created a certain number of specimens of *N* hypothetical species (see Table 1 for default values). Each hypothetical species was assigned a relationship between phenology and climate (i.e., slope; days advanced per unit change in climate) from a normal distribution with the mean of 3 and standard deviation of 1. Each species was assigned a unique mean intercept drawn from a normal distribution with a mean of 268 and a standard deviation of 15. Simulated species were also each assigned an individual flowering duration (i.e., how long an individual of that species takes to progress from buds to fruits) and a flowering season duration (i.e., the period of time within which an individual of that species could progress through its entire individual flowering duration), each drawn from a separate normal distribution with a given mean and standard deviation (values provided in Fig. 2 and Appendix S2).

**Table 1:** Default values for simulated specimen data sets. These values were chosen for their similarity to real‐world values calculated in a study of asteraceous species in the southeastern United States (Pearson, 2019)

Parameter^a^	Default value
Slope (i.e., relationship between phenology and explanatory variable)	3.0
Intercept mean	268
Intercept SD	15
Number of species (*N*)	50
Specimens per species	100
Flowering season duration mean	80
Flowering season duration SD	10
Individual flowering duration mean	20
Individual flowering duration SD	3
Species slope (phenology/climate relationship) mean	3
Species slope (phenology/climate relationship) SD	1

Note

SD = standard deviation.

a The slope and intercept values are arbitrary; changing their values did not affect subsequent results.

Each simulated specimen was randomly designated as one of the *N* simulated species “collected” in climate conditions (i.e., degrees Celsius warmer or cooler than average) drawn from a normal distribution with mean of 0 and standard deviation of 1.2—a value calculated from real‐world temperature deviation data (Pearson, 2019). The simulated peak flowering date for each specimen was drawn from a normal distribution with a mean equal to the intercept of the species and a standard deviation equal to one half of the flowering season duration of that species. The day of collection of each specimen was drawn randomly from a period with a minimum of peak flowering date − ½ × species individual flowering duration and a maximum of peak flowering date + ½ × species individual flowering duration. For the ≥50% method, a subset of simulated specimens was drawn from the full data set, which included only specimens with a day of collection within 25% of the individual flowering duration before or after the peak flowering date.

The observed relationships between phenology and climate were determined from the simulated data as the slopes of linear mixed effects (LME) models that related climate (continuous fixed effect) to either day of peak flowering (for the estimated phenophase method) or day of collection (for the binary and ≥50% methods) while accounting for differences among species (random effect). A second set of models also allowed slopes (i.e., phenology/climate relationship) to vary between species. Model performance was assessed by comparing the width of the 95% confidence intervals (CIs) of the slope calculated using the *confint* function and by comparing the resulting slope values to the predefined mean value. The basic R script for these simulations is provided in Appendix S1.

To evaluate the performance of each method (≥50%, binary, and estimated phenophase) in different circumstances, the following variables were systematically altered, one at a time, while otherwise retaining default values: number of specimens per species, species flowering duration, and individual flowering duration.

### Simulation results

Mean slopes, intercepts, and confidence intervals of all models from all simulations are listed in Appendix S2. For simplicity, statistics provided in this section are for variable‐intercept models only, unless otherwise specified. Corresponding statistics and figures for models in which both slopes and intercepts were allowed to vary are provided in Appendix S3.

In all simulations, the estimated phenophase method produced estimates of slope with significantly narrower confidence intervals compared to both the binary (two‐tailed paired *t*‐test of means of 37 simulations with 100 iterations each: *P* = 4.7e‐5) and ≥50% methods (*P* = 9.7e‐15; Fig. 2). The difference in confidence interval widths was the greatest between the estimated phenophase and ≥50% methods (42–48% difference), whereas the difference between the estimated phenophase and binary methods was often slight (0.3–13% difference) with one notable exception; estimated phenophase models were more robust to longer individual flower durations than binary models (Fig. 2A). Both the binary and estimated phenophase models produced estimates with much narrower confidence intervals than those of ≥50% models, especially when the number of specimens per species was low (Fig. 2B) or the flowering season duration was long (Fig. 2C).

Notably, in the models in which only species intercept was allowed to vary, the average estimated slope did not significantly differ between models using the binary, ≥50%, and estimated phenophase methods (one‐way ANOVA among mean of 37 simulations with 100 iterations each: *P* = 0.98). However, in models with variable species slopes and intercepts, the binary and estimated phenophase methods tended to produce underestimates of slope by 20% and 22%, respectively, while the ≥50% method produced overestimates of slope by an average of 10.6%.

All three methods were equally able to determine the relationship between phenology and the explanatory variable when all 50 species were included in the LME models in these simulations (one‐way ANOVA of slope means for 100 iterations under default conditions: *P* = 0.94). This was also the case, on average, when each species was modeled separately using a simple linear model (*lm* function in R). However, fewer significant (i.e., *P* < 0.05) per‐species relationships were detected when using the binary and ≥50% methods rather than the estimated phenophase method. In a simulation with 100 iterations of the default values (Table 1), an average of 15.8% of per‐species models were significant when using the estimated phenophase method, 15.4% were significant when using the binary method, and only 9.2% were significant when using the ≥50% method.

## CONCLUSIONS

Simulated data suggest that the estimated phenophase method described here can facilitate more precise estimates of phenological relationships than two common, alternative methods. Both the estimated phenophase and binary methods consistently outperformed the ≥50% method by producing up to 48% narrower confidence intervals across all simulations, likely due to the average 50% reduction in sample size produced when using the ≥50% method. Accordingly, the estimated phenophase and binary methods detected a greater number of statistically significant, single‐species relationships of phenophase and the explanatory variable. These results emphasize the importance of acquiring a large enough sample size to detect significant relationships (ideally >100 specimens per species; Fig. 2B), which corroborates previous research (Panchen et al., 2012) and further suggests that excluding all specimens with fewer than 50% of reproductive structures in flower may be unnecessarily restrictive.

The estimated phenophase method outperformed the binary method in all simulations, but this improvement was only slight except in cases when the individual flowering duration was long. In general, as predicted by theory (see Fig. 1), estimated phenological relationships were less precise both with longer species’ flowering durations (Fig. 2C) and longer individual flowering durations (Fig. 2A). This suggests that prior knowledge of a species’ phenological duration is important for focal species selection and can inform the best method by which to assess herbarium specimen phenology. The lack of large model improvement, however, and similar values of slope estimated by all methods also suggest that the somewhat labor‐intensive estimated phenophase method may not be necessary to produce fairly accurate estimates of phenological relationships. Because time and resources are often limited, it may not be feasible to monitor durations of phenological events in the field and perform subsequent analyses necessary to estimate a specific phenophase. Consistent with the findings of Ellwood et al. (2019), the binary method appears to perform remarkably well for species with moderate individual durations of phenological events (10–30 days): a positive outcome for current efforts to digitize specimen‐based phenological data (Yost et al., 2018) and the burgeoning field of specimen‐based phenological research.

## Supporting information


**Appendix S1.** Data and phenological model simulation code.

**Appendix S2.** Mean results of simulated data and phenological models.

**Appendix S3.** Statistics and figures for variable slope and intercept linear mixed effects (LME) models.

**Appendix S4.** R code for assigning phenophase to specimens from proportions of buds, flowers, and fruits.

**Appendix S5.** R code for adjusting “collection date” of herbarium specimens to reflect peak flowering date.

## Figures and Tables

**Figure Figure A1:**
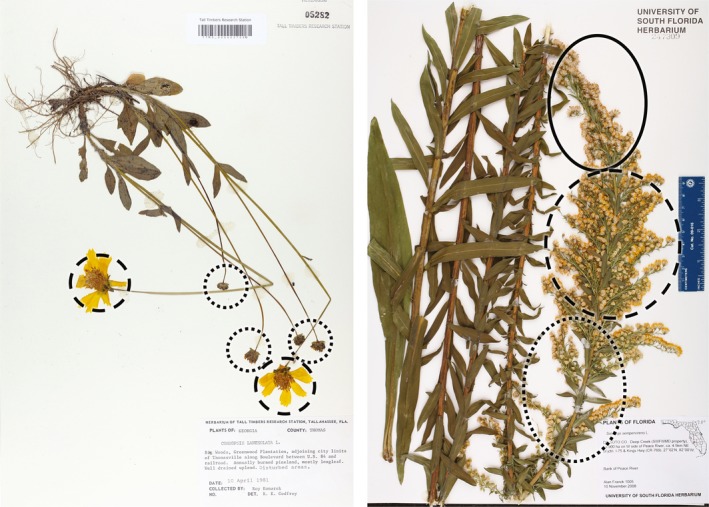
Assessment of bud, flower, and fruit percentages on specimens of the genera *Coreopsis* (left) and *Solidago* (right; Asteraceae). The specimen on the left shows two flowering heads (circled with dashed lines) and four budding heads (circled with dotted lines) and would therefore be coded as 25% flowers, 75% buds—the closest quartile approximation to this ratio. Determining the exact numbers of buds, flowers, and fruits on the specimen on the right is infeasible; however, the relative percentage of each reproductive structure can still be assessed. There are approximately twice as many flowering heads (circled with dashed line) as budding heads (circled with dotted line), and the number of fruiting heads (circled with solid line) and budding heads are roughly equal. Therefore, this specimen would be coded as 25% buds, 50% flowers, and 25% fruits.

**Figure Figure A2:**
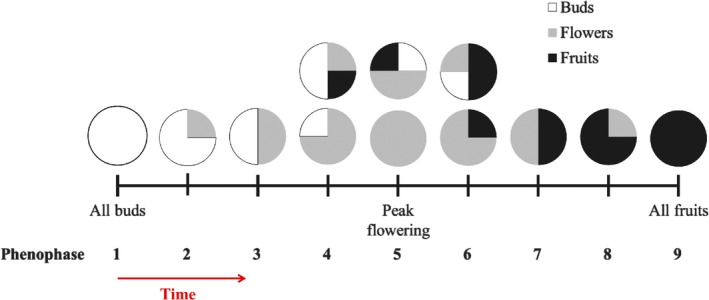
Graphic illustration of assignment of phenophases to specimens based on relative percentages of buds (white), flowers (gray), and fruits (black). Phenophases 4, 5, and 6 can be coded by either of two possibilities because these phases are mutually exclusive yet expected to take place at approximately the same time in the flowering duration.

## Data Availability

R scripts and data created during this research are provided as supporting information accompanying this paper. Additional data are available on the Florida State University digital repository (https://diginole.lib.fsu.edu/).

## APPENDIX 1 Estimated phenophase method protocol.

### Protocol for gathering specimen‐based phenological data

This method requires the evaluator to distinguish between buds, flowers, and fruits on herbarium specimens or images thereof. Because these structures may be difficult to distinguish, especially on small or non‐showy taxa, the evaluator should have a strong botanical background, preferentially with the focal taxa. Experience with living examples of the focal taxa (i.e., field experience) is also strongly encouraged. If multiple evaluators will be scoring specimens, care should be taken to calibrate their phenophase assessments with one another by, for example, using a small set of training specimens.

1. Obtain the sample data set consisting of either physical herbarium specimens or images of those specimens.
2. For each specimen or image, determine the relative percentage of reproductive structures on the sheet that are buds, flowers, or fruits, rounded to the nearest quartile value (0%, 25%, 50%, 75%, or 100%) such that the total of all reproductive structures on a specimen equals 100%. For example, if a specimen has 10 reproductive structures, seven of which are flowers and three of which are fruits, the specimen would be assigned a percentage of 0% buds, 75% flowers, and 25% fruits. In cases wherein there are too many reproductive structures to determine the exact number of buds, flowers, and fruits, estimate the relative percentage to the best of your ability. See Fig. Figure A1 for visual examples.
3. Use the PhenophaseAssignment.R script (Appendix S4) to assign numerical phenophases (1–9) to each specimen record according to the schema shown in Fig. Figure A2.

### Protocol for gathering observational phenological data

1. Locate wild populations of the focal taxa prior to their flowering periods. Ideally, these populations should be genetically diverse, in environmental conditions that are generally representative for the taxon, and unlikely to be severely disturbed (e.g., mowed) during the duration of the observational period, which can last over a month. Ensure proper permitting to conduct monitoring of these populations.
2. In each population, randomly select several (>25) plants that are at or near phenophase 1 (only buds) and that vary in their location within the population, microhabitats (e.g., elevation on the landscape), and any other obvious traits or environmental conditions (e.g., plant height, shading). Clearly label each plant with a unique identifier on a durable tag, such as a metal or waterproof plastic gardening marker.
3. Every 3–4 days (or more frequently, if necessary), determine the relative quartile percentage of buds, flowers, and fruits of each individual plant according to the same schema used to evaluate herbarium specimens. Take a photo of each individual at each visit for future verification. Phenological monitoring of an individual can be stopped when that individual has reached phenophase 9 (100% fruits).
4. When all individuals have reached phenophase 9, remove all tags from the site and cease monitoring.

### Protocol for estimating rate of progression between phenophases and adjusting herbarium specimen collection date

1. Use the PhenophaseAssignment.R script (Appendix S4) to assign numerical phenophases (1–9) to each unique (i.e., individual + day) observational record.
2. Use the first 20 lines of the AdjustCollectionDate.R script (Appendix S5) to determine the rate of transition from one phenophase to another (using the observational data). Ensure that all assumptions of the resulting linear model are met (see Zuur et al., 2010 for guidance).
3. Use the remainder of the AdjustCollectionDate.R code to add or subtract days from the dates of collection of herbarium records to reflect estimated peak flowering dates.
4. Perform desired analyses using the data in the newly created AdjDOY (adjusted day‐of‐year) column.
