# Supplementary material for: A new method and insights for estimating phenological events from herbarium specimens
Source: Appl Plant Sci. 2019 Mar 7;7(3):e01224. doi: 10.1002/aps3.1224 (PMC6426155; doi:10.1002/aps3.1224)
Supplement: Supplementary file 3 — Appendix S3. Statistics and figures for variable slope and intercept linear mixed effects (LME) models. [file APS3-7-e01224-s003.pdf]

DOI

APPENDIX S3. Statistics and figures for variable slope and intercept linear mixed effects (LME) models.

The simulated LME models in which both slope and intercept were allowed to vary among species produced results similar to those of the variable intercept-only models. Results from both model types are provided in Appendix S2, and results from the variable slope + intercept models are described here.

Estimates of slope using the estimated phenophase method had significantly narrower confidence intervals compared to the binary (two-tailed paired *t*-test of means of 37 simulations with 100 iterations each: $P = 4.5e\text{-}5$) and ≥50% methods ($P = 9.1e\text{-}14$; Fig. S3.1). The difference in confidence interval (CI) widths was the greatest between the estimated phenophase and ≥50% methods (24–47% difference) while the difference between the estimated phenophase and binary methods was often slight (0.1–13% difference). Estimated phenophase models were more robust to longer individual flower durations than binary models (Fig. S3.1A). Both the binary and estimated phenophase models produced estimates with much narrower CIs than those of ≥50% models, especially when the number of specimens per species was low (Fig. S3.1B) or the flowering season duration was long (Fig. S3.1C).

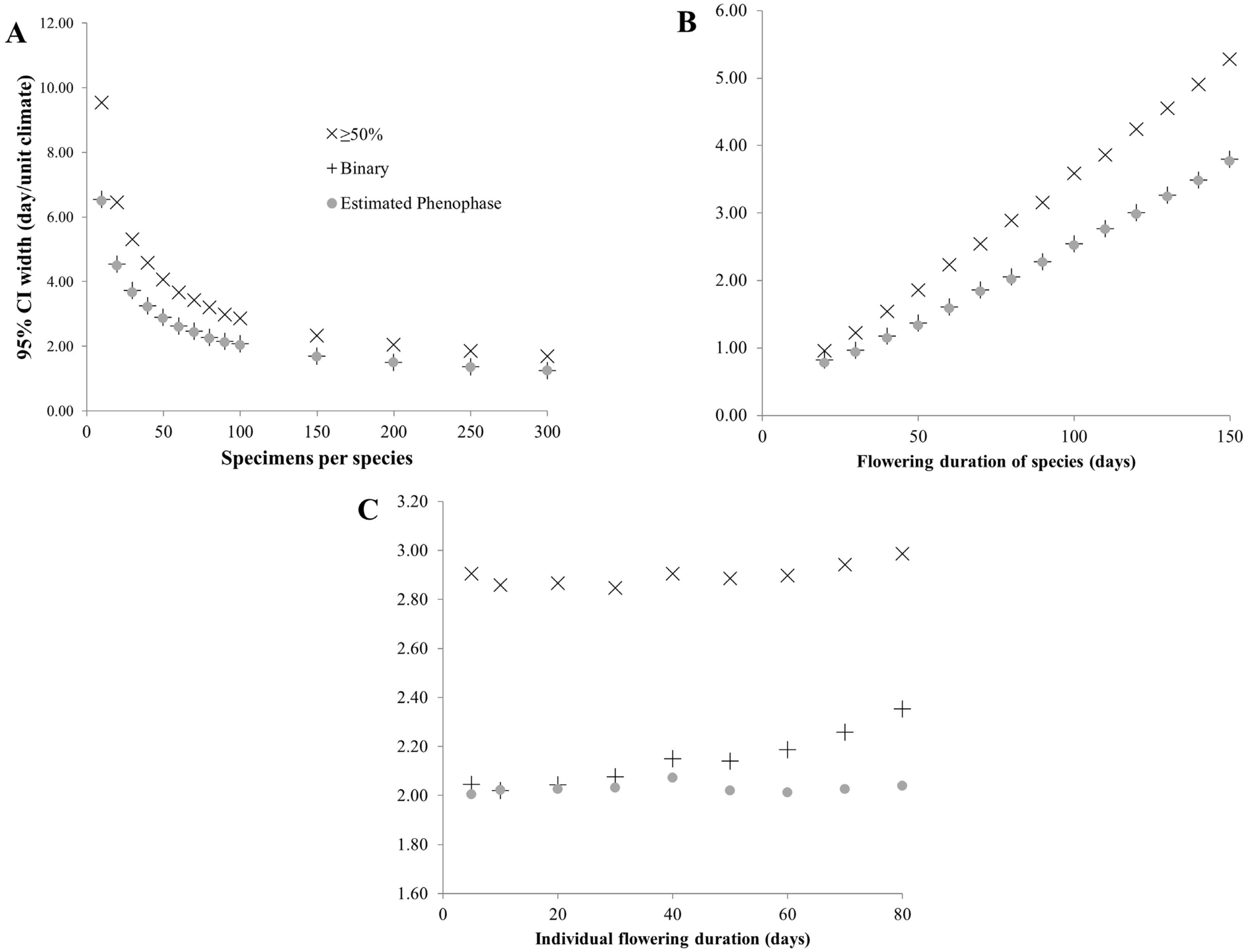

Figure S3.1 Width of 95% CIs of slope (days/unit climate) estimated using the ≥50% (white triangles), binary (gray squares), and estimated phenophase (black circles) methods of specimen inclusion with changes in key simulation parameters. Results shown are those from LME models in which species intercepts and slopes were allowed to vary. Each point represents the mean value of 100 iterations of the simulation. Standard errors of the mean (listed in Appendix S2) were very small and are thus not included in this figure, for clarity. (A) The length of the individual flowering duration was changed between simulations. (B) The number of specimens per species was varied while keeping all other variables, including number of species, constant. (C) 95% CI widths with increasing flowering season durations of species. Unless otherwise specified, default simulation parameters were as described in Table 1.
